# Leishmaniasis and glycosaminoglycans: a future therapeutic strategy?

**DOI:** 10.1186/s13071-018-2953-y

**Published:** 2018-10-03

**Authors:** Débora Almeida Merida-de-Barros, Suzana Passos Chaves, Celso Luis Ribeiro Belmiro, João Luiz Mendes Wanderley

**Affiliations:** 1 Laboratório de Imunoparasitologia, Unidade Integrada de Pesquisa em Produtos Bioativos e Biociências, Campus UFRJ-Macaé, Universidade Federal do Rio de Janeiro, Rio de Janeiro, Brazil; 2 Programa de Pós Graduação em Produtos Bioativos e Biociências, Campus UFRJ-Macaé, Universidade Federal do Rio de Janeiro, Rio de Janeiro, Brazil

**Keywords:** *Leishmania*, Glycosaminoglycans, Macrophage, Receptors

## Abstract

*Leishmania* spp. depend on effective macrophage infection to establish and develop in mammalian hosts. Both metacyclic promastigotes and amastigotes are able to infect host cells, and thus they rely on several ligands that, when recognized by macrophage receptors, mediate parasite uptake. During macrophage primary infection with metacyclic forms from the insect vector and during amastigote dissemination *via* macrophage rupture, both infective stages have to cope with the host extracellular microenvironment, including extracellular matrix molecules. Glycosaminoglycans are abundant in the extracellular matrix and many of these molecules are able to interact with the parasite and the host cell, mediating positive and negative effects for the infection, depending on their structure and/or location. In addition, glycosaminoglycans are present at the surface of macrophages as proteoglycans, playing important roles for parasite recognition and uptake. In this review, we discuss glycosaminoglycans in the context of *Leishmania* infection as well as the possible applications of the current knowledge regarding these molecules for the development of new therapeutic strategies to control parasite dissemination.

## Background

Innate immune cells express pattern recognition receptors (PRRs) on their surface, and they recognize pathogen-associated molecular patterns (PAMPs). Important examples of such receptors are dectin receptors, complement receptors (CR), mannose receptors (MR), scavenger receptors and Toll-like receptors (TLR). Several pathogens use these receptors as a route for cellular infection including protozoan parasites of the genus *Leishmania*. These parasites are the causative agents of leishmaniasis, a vector-transmitted disease endemic in several countries, especially in tropical and subtropical regions [1].

*Leishmania* parasites have as their main target cell the macrophage, a phagocytic cell that richly expresses PRRs. Infective forms of promastigotes and amastigotes of *Leishmania* use phagocytic PRRs as a way to interact with and infect host macrophages [2]. In addition, this interaction is capable of differentially activate PRRs to modulate and evade the immune response [3]. On its surface, *Leishmania* parasites express molecules that promote their binding to macrophages. One of them, glycoprotein 63 (gp63), is responsible for cleaving the C3b complement protein in iC3b [4], which opsonizes and binds the promastigote to the complement receptor 1 (CR1) [5]. Gp63 can also directly bind to fibronectin receptors [6]. Another surface molecule that induces *Leishmania* phagocytosis is the lipophosphoglycan (LPG), which binds to mannose receptors in macrophages [7] and complement receptor 3 (CR3) [8]. In *Leishmania infantum chagasi*, non-virulent promastigotes have been reported to bind to CR3 and mannose receptor, whereas metacyclic forms bind primarily to CR3. The entry *via* CR3 promotes a delay in phagolysosomal fusion, promoting parasitic survival [2]. Regarding amastigotes, an important route of entry into the cell is through IgG opsonization *via* Fcγ receptors [9] and through the exposure of phosphatidylserine (PS). Amastigote forms expose PS on their surface, mimicking apoptotic bodies, which leads to the release of TGF-β by infected macrophages leading to a decrease in nitric oxide (NO) production [10]. Additionally, macrophage recognition of PS molecules induces tethered and bystander macropinocytic uptake of particles, which leads to amastigote internalization and infection establishment [10]. This mechanism is also important for metacyclic primary infection, but in this case, the parasites are actually suffering apoptosis, and this is important for phagocyte deactivation and the establishment of live, PS-negative metacyclic promastigotes, including in natural transmission [11].

There are also PRRs that *Leishmania* uses to modulate infection and/or evade the immune response, for example, Toll-like receptors [3]. Recently, Dos Santos et al. [12] demonstrated that the activation of human nucleotide-binding oligomerization domain containing 2 (NOD2)-like receptor (NLR) is important for the intracellular recognition and control of New World *Leishmania* spp., etiological agents of American tegumentary teishmaniasis. In addition to receptor activation, *Leishmania* has several mechanisms of immune response evasion. The transformation into the intracellular amastigote is a form of resistance to the immune response, since it is more resistant to the low pH of the parasitophorous vacuole, oxygen peroxide, nitric oxide and lysosomal enzymes. Gp63 and LPG mediate mechanisms already described, where both have complementary actions. After endocytosis, LPG delays the fusion of the phagosome with the lysosome and gp63 inhibits the activation of lysosomal enzymes, favoring the survival of *Leishmania* [13–15]. In addition, both molecules are capable to inhibit the oxidative burst [16, 17]. *Leishmania* infection leads to the interruption of several macrophage effector mechanisms, such as reduction of the microbicidal capacity by inactivation of the enzyme inducible nitric oxide synthase (iNOS) and oxidative system [18]. PGE2 and TGF-β release block the function of macrophages [19]. The C3b cleavage in iC3b by Factor I, induced by gp63, helps in the avoidance of the effector actions of the complement system [19]. There are also evasion mechanisms of the adaptive immunity, such as induction of decreased expression of MHC class II molecules [20], inhibition of antigen loading and processing, and induction of Th2 response by Lack antigen.

It has also been shown that *L. amazonensis* promastigotes possess ecto-ATPases, which have been related as virulence factors of the parasite. During tissue injury, release of ATP to the extracellular medium occurs, inducing the production of inflammatory cytokines such as IL-12 and TNF-α. These ecto-ATPases degrade ATP in ADP and later in AMP. This is converted into adenosine by CD73, which has an anti-inflammatory role in the infection by decreasing the production of inflammatory cytokines and stimulating IL-10 release [21, 22].

Although several different receptors and ligands play major roles in *Leishmania*/macrophage interactions, usually the infection is not completely abrogated by the blockage of one or some of these mechanisms of infection. This indicates that there are other players that have not been fully identified yet, influencing the infective process. In studies of parasite/host relationship, it has been demonstrated that proteoglycans and glycosaminoglycans are also involved in modulating macrophage infection by the promastigotes and amastigotes of *Leishmania* spp. as well as modulating the infected host cell itself [23–25].

## Proteoglycans

Proteoglycans (PGs) are a heterogeneous group of glycoconjugates widely distributed in animal tissues, from the earliest to the most recent phyla [26]. All PGs identified are composed of glycosaminoglycan chains (GAGs) covalently attached to a protein skeleton [27, 28]. These macromolecules present a great structural diversity due to the many possibilities of polysaccharide and protein binding, different proteins involved, and GAGs’ structural diversity. This characteristic contributes to the involvement of PGs in a wide variety of biological functions [29, 30]. The PG superfamily contains more than thirty known molecules. They act as tissue organizers, influence the growth and maturation of specialized tissue cells, function as biological filters, modulate the activity of growth factors, modulate inflammatory responses, regulate collagen fibrillogenesis, affect cell invasion and growth and are involved in host-parasite relationships [28, 31–33].

With the exception of hyaluronic acid (HA) all other GAGs are synthesized as PGs. This process begins in the rough endoplasmic reticulum with the addition of a xylose residue to the amino acids serine or threonine in the protein skeleton [30, 34]. Then, specific transferases of the cis and median portions of the Golgi complex add two units of galactose and one of glucuronic acid, forming the binding tetrasaccharide, common to most GAGs [35, 36]. In the case of keratan sulfate (KS), GAG chains may be attached to the protein *via O*- or *N*-glycosidic *N*-acetyl-galactosamine (GalNac) or *N*-acetyl-glucosamine (GlcNAc) to the serine or asparagine/threonine residues, respectively. The binding of the tetrasaccharide functions as an initiator sequence for the polymerization of the GAG chain in the biosynthesis of chondroitin sulfate, dermatan sulfate, heparan sulfate and heparin [35, 36]. Chain elongation occurs through the alternating addition of hexosamine and hexuronic acid by membrane-specific glycosyltransferases. After polymerization, the GAG chain is modified by the action of epimerases, that determines which glucuronic acid units will be transformed into iduronic acid, and by sulfotransferases, which determine the degree of sulfation of the final structure that is often transported to the cell surface or extracellular matrix [27, 31–33].

### Classification of proteoglycans

PGs can be classified according to the following parameters: type of GAG, cell location and homology of their protein skeleton [32]. The first parameter is outdated, since the same type of PG can present different types of GAG chains. Here we briefly describe the different families of PGs classifying them according their cellular or extracellular location.

#### Extracellular proteoglycans

Versican, aggrecan, neurocan and brevican are PGs secreted and deposited in the extracellular matrix and play a very important role filling intercellular spaces and keeping cells and tissues together. These PGs interact with hyaluronic acid (HA) and lectins, also known as hyalectans [32]. The common characteristic is the presence of three well-defined domains: the N-terminal domain, which binds hyaluronic acid; the repetitive central domain, which contains GAG chains of the type chondroitin sulfate (CS); and KS, and the C-terminal domain, which binds to lectins [37]. These features allow these PGs to function as molecular bridges between matrix components and the cell surface [28, 31–34].

Small leucine-rich PGs are typically characterized by having core proteins with leucine rich sequences. The protein skeleton of these PGs has three distinct regions: an amino-terminal containing GAGs or L-tyrosine sulfate, a central domain containing leucine-rich repeating sequences bounded by residues of L-cysteine and a carboxy-terminal region that is not well characterized [28]. To date, at least nine families have been characterized, based on their genomic and protein organization. As examples we have decorin and biglycan, both PGs containing chondroitin sulfate and/or dermatan sulfate chains [31, 33].

Facultative PGs do not have structural characteristics that include them as members of the families previously described. In addition, they can be found as an exclusive constituent of proteins expressed in a GAG-free chain form. Collagen 2 (IX), testican and apican are examples of PGs of this group [31–33].

Basement membrane PGs are extracellular PGs found in the basal lamina. They form a thin, flexible layer of specialized extracellular matrix located under all cell layers [32]. In the pulmonary alveoli and renal glomeruli, the basal membrane functions as a selective filter but it can have different functions in other organs [38]. Base membrane PGs present a variable composition among the tissues, but the presence of collagen type IV, laminin and at least one type of PGs from other families is common [31–33].

#### Proteoglycans of cytoplasmic granules

PGs can be found inside cytoplasmic granules, such as serglycin which is a constituent of mast cells, neutrophils and endothelial cells granules. These PGs are important for the binding and storage of proteases within the secretory cytoplasmic granules. They are involved with inflammatory and immune responses. Their nomenclature depends on the protein chain and the number of repetitive sequences of L-serine-L-glycine [31–33].

#### Cell surface proteoglycans

All processes of cell communication and interaction, both with other cells or extracellular matrix components, can be attributed, at least in part, to the surface PGs. Most animal cells have on their surface PGs that may contain mostly heparan sulfate chains, but also chondroitin sulfate and dermatan sulfate [39]. Association with cell surfaces can occur in several ways: directly through non-covalent interactions between the GAG chain or the protein chain with binding sites present on the cell membrane; by intercalation of the PG protein chain in the membrane; by the anchoring the PG to the membrane *via* glycosylphosphatidylinositol (GPI) lipid anchors [40, 41] or indirectly involving other matrix macromolecules such as laminin or fibronectin, which are capable of binding PG and integrins [31–33, 42]. Glypicans, are GPI anchored heparan sulfate-based PGs which has been identified in several cell types [37, 39]. Syndecans are PGs inserted into the plasma membrane through a hydrophobic domain, which are mainly found in epithelial cells. Syndecans 1 and 3 present hybrid chondroitin sulfate and heparan sulfate chains whereas syndecans 2 and 4 contain only heparan sulfate. Among the functions attributed to this family of PGs, most are related to the ability to recognize extracellular molecules since syndecans bind to various molecules, such as collagens type I to V, fibronectin, thrombospondin, tenascin and laminin [39]. In addition, they bind to molecules that are associated with cell growth such as bFGF, VEGF, HGF and some cytokines [34, 43–47]. Other PGs, also integral membrane molecules are: CD44, betaglycan (TGF-growth factor receptor), transferrin receptor, invariant chain PGs, integrin 51 and thrombomodulin [31–33, 42, 48].

## Glycosaminoglycans

**Fig. 1:**
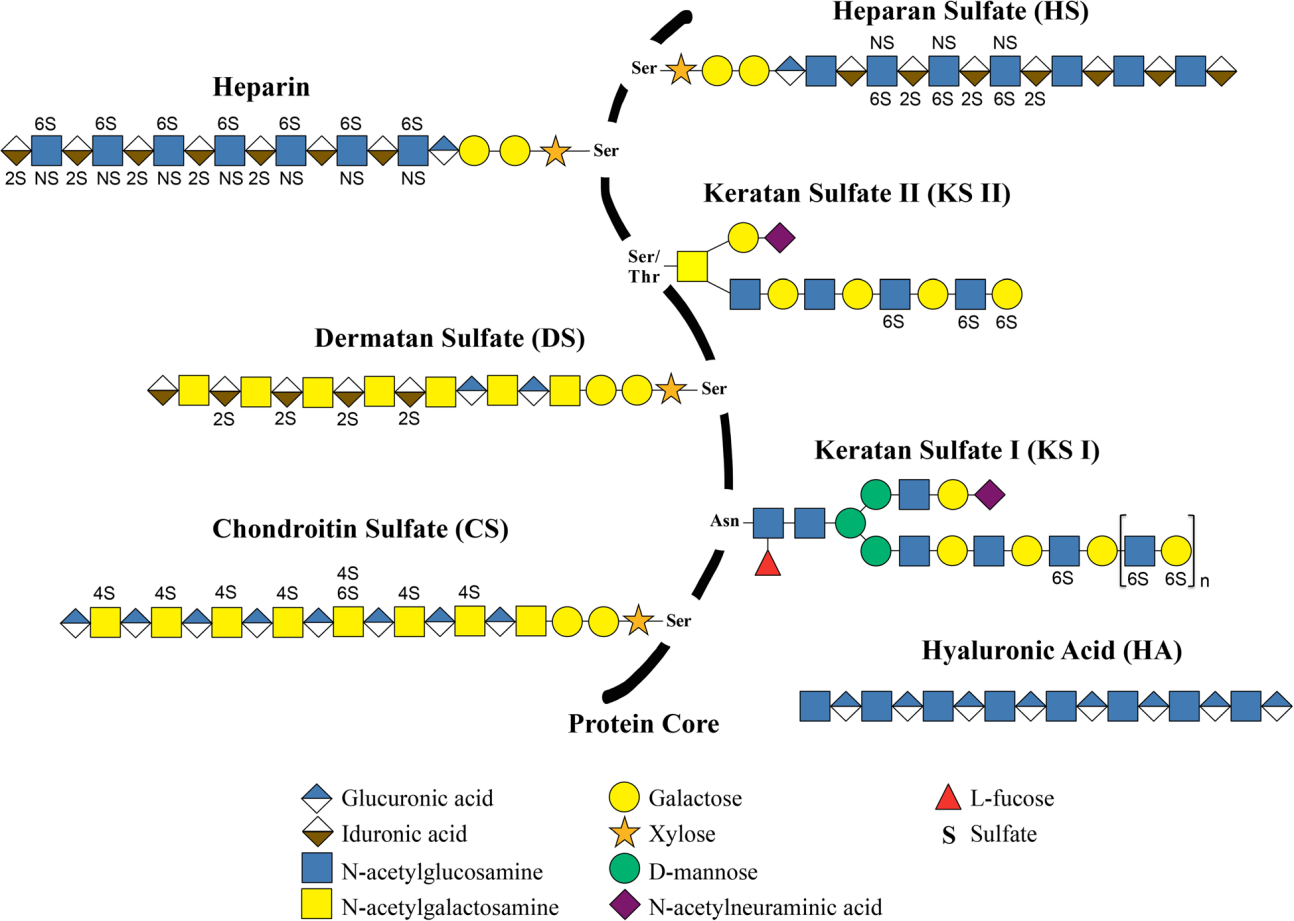
Schematic representation of glycosaminoglycan chains. Heparan sulfate, heparin, chondroitin sulfate, dermatan sulfate, keratan sulfate I and keratan sulfate II are represented bound in a protein core, therefore as proteoglycans. Hyaluronic acid is represented in an isolated form since it is not synthesized inserted into a protein core. Adapted from [32]

Glycosaminoglycans (GAGs) are a complex family of linear polysaccharides, consisting of repeating disaccharide units of hexosamine (*N*-acetyl-glucosamine or *N*-acetyl-galactosamine) attached through *O*-glycosidic linkage to a hexuronic acid (glucuronic acid or iduronic acid) or to galactose [37]. Hexuronic acid residues may exhibit *O*-sulfation at carbon 2, while galactose may be 6-*O*-sulfated. However, hexosamine may exhibit *O*-sulfation at carbons 4 and/or 6 and also *N*-sulfation [37]. The structure of different GAGs is depicted in Fig. 1. Sulfate groups, together with the carboxylic acids of uronic acids, attribute to these molecules a high density of negative charges. In recent years, studies of polysaccharides, especially those related to their biological activities, have been attracting increasing interest. The applicability of this group of molecules in different fields, such as health, industry and agriculture, is due to structural characteristics, often species-specific, associated with heterogeneity in size, monosaccharide composition and presence of different negatively charged groups, such as sulfates and carboxylates [42, 49]. Table 1 summarizes studies on different GAGs and their main functions [50–73].

**Table 1:** References regarding the physiological functions of glycosaminoglycans in animal cells and tissues

Physiological function	Heparin	Heparan sulfate	Chondroitin sulfate	Dermatan sulfate	Hyaluronic acid	Keratan sulfate
Cell-cell/cell-matrix interactions	–	Thakar et al. [54]	Milstone et al. [58]	Lewandowska et al. [63]	Underhill et al. [67]	Funderburgh et al. [72]
Immune modulation	Almeida et al. [50] Borsig et al. [51]	Almeida et al. [50]; Thakar et al. [54]	Borsig et al. [51]	Nadafi et al. [64]	Horton et al. [68]; Termeer et al. [69]	Leroux et al. [73]
Host-pathogen interactions	Angeletti et al. [52]	Takemae et al. [55]; Ndonwi et al. [56]	Rogerson et al. [59]; Ayres Pereira et al. [60]	Ndonwi et al. [56]; Fortune et al. [65]	Beeson et al. [70] Skinsnes et al. [71]	–
Anticoagulant activities	McLean et al. [53]	Shworak et al. [57]	Mourão et al. [61]; Glauser et al. [62]	Ofosu et al. [66]	–	–

### Hyaluronic acid (HA)

HA has a simple structural composition, being fundamental in the structural organization of the extracellular matrix. Its disaccharide units, composed of *N*-acetylglucosamine and glucuronic acid are not sulfated, therefore the anionic properties of HA are given by its carboxyl groups [37]. Unlike other GAGs, HA is not synthesized covalently linked to the protein chain, and is therefore classified simply as GAG [74]. They form high molecular weight polymers ranging from 106 to 107 kDa, being important components of the extracellular matrix, where they interact non-covalently with the PGs. They are particularly abundant in the connective tissue, dermis, smooth muscle, lung, lamina propria of the mucosa and adventitious layer that surrounds the blood vessels [42, 49, 74].

### Chondroitin sulfate (CS)/dermatan sulfate (DS)

CS and DS are considered as GAGs belonging to the same family because they present as disaccharide units an *N*-acetylgalactosamine linked to a glucuronic acid in the case of CS or to an iduronic acid in the DS case. CS can have the sulfated *N*-acetylgalactosamine at the 4- or 6- positions, forming the chondroitin 4- or 6-sulfates, respectively. DS is an isomeric form of chondrocyte 4-sulfate where, in most of its disaccharide units, we find iduronic acid instead of glucuronic acid. The sulfation pattern of these GAGs may still be very complex because, depending on the type of tissue or organism, the disaccharide units may exhibit extra sulfation giving rise to the disulfated disaccharide units [37, 75]. In the case of CS, the *N*-acetylgalactosamine can be both sulfated at its 4- and 6- positions whereas in DS the extra sulfation can be at the 2-position of iduronic acid [42, 49].

### Keratan sulfate (KS)

KS has disaccharide units composed of *N*-acetylglucosamine, which can be sulfated at the position 6, and galactose instead of a hexuronic acid. In addition to this particularity, KS chains can be either *O*- or *N*-linked to the PG chain, having as the point of attachment a serine or asparagine/threonine residues, respectively. We can cite two types of Keratan sulfate according to their binding to protein core, KS type I and KS type II [37, 42, 49, 76].

### Heparin (Hep) and heparan sulfate (HS)

Both Hep and HS present the glucosamine amino sugar, which may be *O*-sulfated at position 6 in the case of HS and preferably *N*-sulfated at the 2-position and *O*-sulfated at position 6, in the case of heparin. Regarding the composition of hexuronic acid, it is verified that heparin contains mainly iduronic acid, with varying amounts of *O*-sulfation at position 2, whereas HS contains more glucuronic acid, usually not sulfated. However, it is important to note that there are differences between these two molecules. The degree of sulfation is one of the main factors used to differentiate these two molecules. Based on this information, it is possible to say that heparin is composed mainly of disaccharide units of type IdoA(2S) GlcNS(6S), while HS contains a small proportion of this disaccharide, being mainly characterized by having a greater variety in composition of their disaccharide units. However, these are less sulfated than those present in heparin. It is important to note that heparin can be isolated from different animal tissues, including vertebrates or invertebrates [26, 77]. In mammals, it is synthesized and stored in cytoplasmic granules of mast cells, being released as free non-protein bound molecules after activation and degranulation of mast cells, in events associated with the immune system. HS is synthesized and expressed on the cell surface of a wide variety of cell types, and can be secreted either in the form of free GAGs or PGs [34, 48, 49, 78, 79].

### Occurrence and biological activities of glycosaminoglycans

The presence of GAGs in vertebrate and invertebrate animal cells and tissues is already well described in the literature [31, 33, 80]. Research has shown that these GAGs are involved in several biological functions such as cell-cell and cell-to-matrix interactions [33, 81], immobilization of cytokines and chemokines, cell adhesion and in the immune response of different types of organisms [81–84]. These molecules also play a role in host-parasite interactions [85], in addition to the known remarkable anticoagulant activities of these molecules [80]. Among these biological activities we will focus on host-parasite interaction. In several cases, the ability of pathogenic microorganisms to bind to the surface of host cells, define the course of infection. In this sense, a wide variety of bacteria, viruses and parasites, including intracellular and extracellular ones, was identified, which require interaction with host cell heparan sulfate molecules *via* a variety of HS receptors to establish their infections [85–87].

In recent years, studies of GAGs, especially those related to their biological activities, have been attracting significant interest. The applicability of this group of molecules in distinct fields, such as health sciences, is due to structural characteristics, often species-specific, associated with size heterogeneity, monosaccharide composition and the presence of different negatively charged groups such as sulfates and carboxylic acids [88, 89]. In recent decades, GAG analogues have been identified, especially in marine organisms such as the ascidian, sea cucumbers, mussels, crustaceans and mollusks [29, 90]. Studies have shown that these analogues have many biological activities [49]. GAGs were isolated and characterized as mussel heparin analogs with significant anticoagulant activity [90]. A heparin analogous to the mammalian heparin was isolated and characterized from the ascidian *Styela plicata*, presenting less anticoagulant activity, although with anti-inflammatory activity [49, 89]. In addition, the identification of a heparan sulfate-like molecule from the mollusk *Nodipecten nodosus*, revealed a molecule with potent anticoagulant activity, capable of inhibiting thrombus formation in ischemic diseases [91] A dermatan sulfate with a high degree of sulfation was also identified and characterized in *Styela plicata* and *Phallusia nigra* ascidian, with specific anticoagulant activities. In addition, the development of ascidian neural cells has also been associated with these GAGs [92, 93]. Furthermore, chondroitin sulfate analogues from sea cucumbers *Ludwigothurea grisea*, have antithrombotic activity, in addition to inhibiting the binding of P-selectin to leukocytes [94, 95].

An important aspect to consider when proposing the therapeutic use of natural compounds of animal origin is the risk of contamination with pathogens. For example, the association of prion mammalian proteins with transmissible spongiform encephalopathy has recently restricted the use of bovine heparin in Europe, Japan and the USA [96]. Currently, commercial heparin is obtained exclusively from swine tissues and the risk of contamination is still present. Therefore, when considering therapeutic strategies using mammalian GAGs, it is important to take account their side effects and the possibility of contamination with pathogens. In this context, the search for alternative analog GAG compounds obtained from non-mammalian animal sources and having similar biological activities, becomes extremely relevant.

## Glycosaminoglycans and *Leishmania* infection

### Glycoconjugates and *Leishmania* infection

Natural *Leishmania* infection involves several differentiation steps, culminating with the development of infective metacyclic promastigotes that migrate to the sand fly anterior midgut [97, 98]. The parasites remain there embedded in a filamentous proteophosphoglycan matrix called promastigote secretory gel (PSG) that jams the food flow blocking a new blood intake. This is fundamental for the transmission, since the vector needs to regurgitate the content of the gut to take a new blood meal, delivering parasites, saliva and PSG intradermally to the mammalian host [99–101]. Actually, *Leishmania* parasites are prone to produce phosphoglycan-containing molecules with variably phosphodisaccharide repetitions, predominantly (Gal-Man-PO_4_). These molecules include the lipophosphoglycan (LPG), PSG and proteophosphoglycans [102]. Whereas LPG is a membrane-anchored molecule that functions as a virulence factor, being recognized by complement receptors in phagocytic cells [8], PSG is secreted and seems to be involved in the modulation of the microenvironment at very early stages post transmission. PSG is actively expelled by infected sand flies during the blood meal and its presence exacerbates cutaneous infection in both resistant and susceptible mice strains [100, 101]. This effect seems to be due to the chemoattraction of macrophages and neutrophils to the lesion site, providing host cells for the establishment of the infection [103]. In addition, PSG is able to reduce the leishmanicidal activity of M1 macrophages, through induction of arginase I expression in these cells and thus diminishing nitric oxide production [103]. Combined, the consequences of PSG promote the development of chronic *Leishmania* infections, even in resistant mice strains, in a LPG-independent and proteophosphoglycan-dependent manner [100]. This mechanism operates in a large variety of *Leishmania* species such as *L. mexicana*, several strains *of L. major*, *L. amazonensis*, *L. braziliensis*, *L. aethiopica*, *L. infantum* and *L. donovani* [100]*.* Although the effects of PSG seem to be as important as the sand fly saliva to enhance and modulate infection, the receptors involved on its recognition in both parasite and host cells are largely unknown.

*Leishmania* parasites and other protozoan parasites possess immunogenic glycoconjugates at their surface. Most of these molecules are glycosylphosphatidylinositol (GPI) anchored molecules, similar to glypicans, although they are not considered GAGs due to differences in structure and synthesis [104]. These molecules are collectively known as glycoinositolphospholipds (GIPLs) and have a basic structure of Manα1-4GlcN linked to an alkyl-acylglycerol through a phosphatidylinositol residue [105]. Polymorphic and biochemical variations led to the classification of these molecules in three groups: Type I, II and III GIPLs. Type I GIPLs are composed of an α1,6-mannose residue linked to Manα1-4GlcN motif. This type of GIPL is observed in Old World *Leishmania* species such as *L. donovani*, *L. aethiopica* and *L. tropica* [106]. Type II GIPL presents an α1,3-mannose residue linked to Manα1-4GlcN motif and is structurally associated to LPG molecules. It is widely observed in Old World and New World *Leishmania* species such as *L. major*, *L. panamensis* and *L. mexicana* [107–109]. Type III GIPLs are heterogeneous molecules with mixed structural characteristics of type I, type II and unclassified GIPLs [109]. GIPLs are highly immunogenic and leishmaniasis patients are usually positive for specific antibodies anti-GIPL, although there is no described correlation with protection. This is particularly demonstrated in *L. major*-infected patients [104], and this characteristic is being explored in a vaccine-based therapeutic strategy based on αGal-containing neoglycoproteins [110]. These molecules act as virulence factors since they seem to negatively regulate macrophage activity as observed in *L. major*, *L. braziliensis* and *L. infantum* infections [111].

**Fig. 2:**
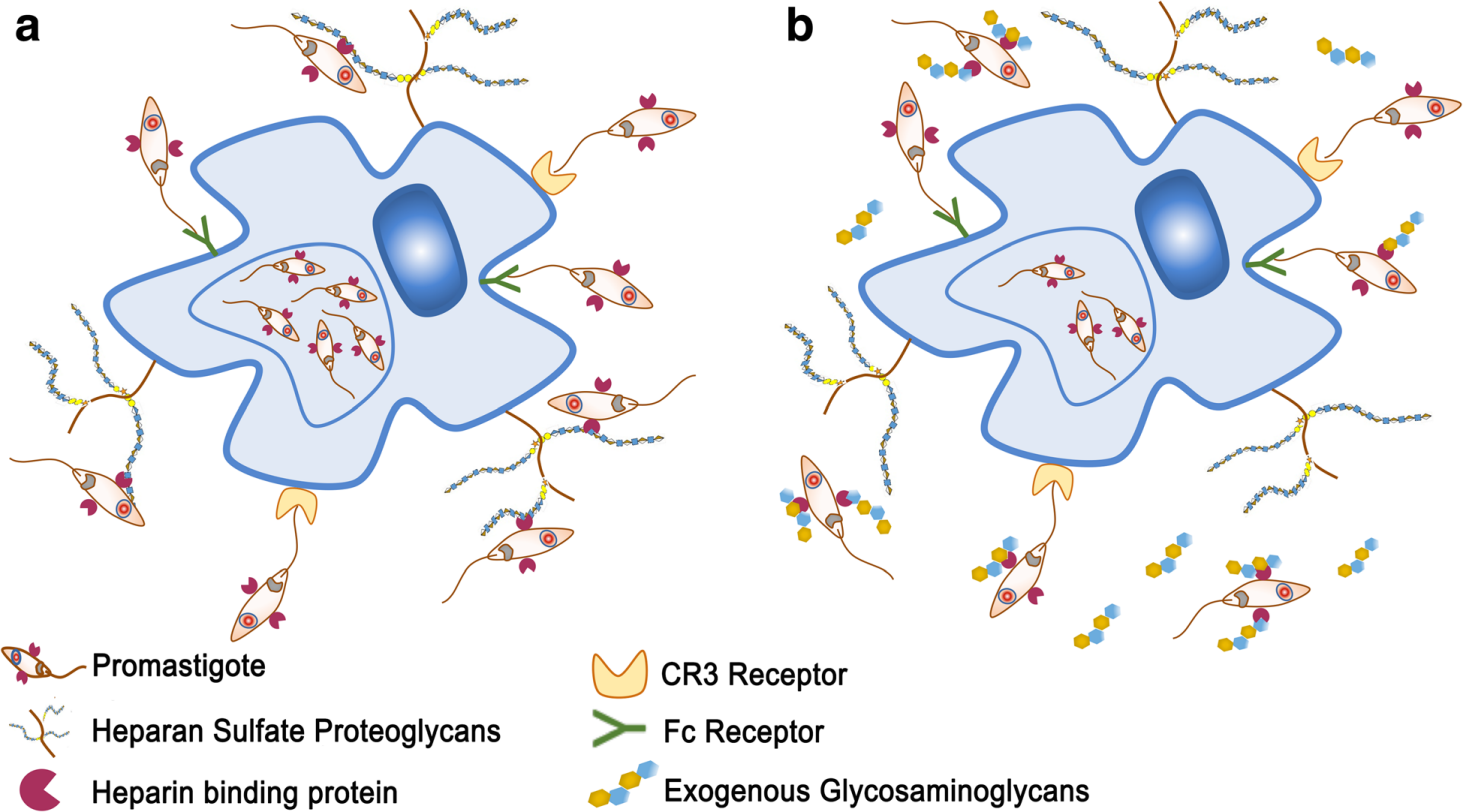
Glycosaminoglycans as a therapeutic strategy. **a** Heparin-binding protein from *Leishmania* spp. recognizes and binds to glycosaminoglycans (GAGs) in the surface of macrophages. This recognition is important for parasite binding to and infection of macrophages. **b** Exogenous GAGs may interfere with this interaction, decreasing macrophage infection and parasite dissemination/survival

Once transmitted by an infected sand fly, parasites have to cope with the host microenvironment, especially the extracellular environment, which is not their natural residence. Flagellate promastigotes and amastigotes, the infective stages of the parasite, are deposited in the lesion site extracellular matrix, respectively during the blood meal and macrophage rupture. During these events, parasites interact with extracellular matrix (ECM) proteins, sugars and growth factors [112–114] and these interactions are important for infection establishment. In addition, macrophages and other host cells interact with ECM contents and these interactions have positive and negative effects for the infection [115]. In this context, GAGs are interesting molecules because they are able to interact with parasites when encountered in ECM and at the surface of host cells modulating parasite growth and lesion development [115]. Parasite interaction with surface PGs or exogenous GAGs is depicted in Fig. 2 and is discussed in this review. GAGs’ interactions with parasites were first evaluated by treating *L. donovani* promastigotes with hyaluronidase or hyaluronic acid (HA) to observe *in vitro* growth, viability and motility. It was observed that hyaluronic acid treatment induced marked reduction of promastigote mobility, without evidence of toxicity, suggesting that the presence of this molecule can facilitate parasite uptake by host cells. However, at that point there was no evidence of direct binding of HA to the surface of promastigotes [116]. It has been shown that heparin is capable of modulating protein kinase activity, both positively and negatively [25]. Since heparin can be present in the lesion site and be produced by resident leukocytes involved in inflammatory responses such as mast cells, its effect on promastigotes of *L. donovani* was observed. Heparin is able to inhibit protein kinase activity of promastigotes, decreasing the amount of phosphorylated proteins. This activity is dependent on heparin-binding to promastigotes *via* specific receptors, since dermatan sulfate (DS), chondroitin sulfate (CS) or HA were not capable of competing with heparin for its receptor [25]. Direct effects for parasite infectivity of protein kinase inhibition due to heparin-binding was not observed. The heparin receptor of the parasite is a surface protein, directed to the plasma membrane from an intracellular stored pool, mainly expressed at the flagellum and flagellar pocket [23]. Its specificity depends on the size of the oligosaccharide chain, with at least 8–16 monosaccharide repetitions being necessary for proper binding. The level of sulfation is also important for promastigote binding to GAGs. Complete desulfation of octa-, dodeca- and hexadecasaccharides almost completely abrogates promastigote binding to these GAGs. On the other hand, oversulfation of DS is able to promote promastigote binding to this GAG [23]. In a more detailed work, Fatoux-Ardore et al. [113] evaluated 24 different strains from six different species of *Leishmania* for their interactions with ECM GAGs and proteins. They analyzed Old and New World species that cause cutaneous, mucocutaneous or visceral leishmaniasis. The capacity of promastigotes to bind to GAGs was assessed by surface plasmon resonance imaging and it was observed that most strains tested are capable to bind to high molecular weight heparin, heparan sulfate, 2-*O*, 6-*O* and *N*-desulfated heparin, whereas less than 12% of the strains tested were capable to bind to dermatan and chondroitin sulfate. Binding to low and high MW hyaluronan was observed by most species tested. However, few parasite strains were able to bind to these molecules, with the exception of *L. donovani* promastigotes. It is interesting to observe that several strains that were able to bind to heparin do not bind to HS [113]. This can be due to the level of sulfation observed in these molecules and is relevant for possible correlations between heparin-binding ability of promastigotes and the HS proteoglycan-dependent macrophage infections. One possible role for GAGs interactions with *Leishmania* parasites is that these molecules are able to induce the activity of cysteine proteinases. Parasites in the ECM prior to host cell engulfment also interact with ECM proteins and can co-opt some of these molecules for their growth, survival and infection. GAGs can form ternary complexes with cruzipain and cathepsin K and B, increasing kininogen and collagen proteolytic cleavage [117–120]. GP63, which is the main surface protease of *Leishmania* parasites, is also important for ECM cleavage [112]. GAGs also modulate cysteine proteinase B (CPB), an enzyme important for *L. mexicana* infection, nutrition and immune system escape [121]. Heparan, heparin and heparin-like GAGs were able to modulate CPB activity. This modulation can be positive and negative since heparin at concentrations below 2 mM can potentiate the production of mature CPB whereas at concentrations above 20 mM these molecules inhibit this conversion [121]. It is possible to conclude that the interaction with host cells and the lower concentration of GAGs associated with proteoglycans could induce CPB activation, contributing to the infectious process, while the higher concentrations of free chains of GAGs at the ECM at the inflammatory site of the infection could contribute to CPB inhibition and parasite control. ECM GAGs can also have positive effects for the infection, stimulating parasite survival. Intracellular amastigote forms of *L. major* depend on the uptake and degradation of ECMs GAGs by macrophages [115]. *N*-acetylglucosamine acetyltransferase (GNAT) deficient *L. major* amastigotes are not capable to proliferate inside *in vitro* cultured macrophages, even in the presence of *N*-acetylglucosamine (GlcNAc). Amastigote proliferation is only efficient *in vivo* or *in vitro* when macrophages are cultured in media enriched with HA which is a desulfated GlcNAc-rich GAG abundant in the ECM [115]. It was observed that HA is engulfed by macrophages, transported to the parasitophorous vacuoles and degraded. The GlcNAc residues are used by the parasite to resume production of glycoconjugates, including plasma membrane glycolipids and proteoglycans including lipophosphoglycan and GP63, important virulence factors [115].

### Modulation of *Leishmania*/macrophage interaction by glycosaminoglycans

Direct binding of *Leishmania* parasites with cell surface proteoglycans has been shown to play a role for the infection of *L. amazonensis*, *L. major*, *L. donovani*, *L. infantum chagasi*, *L. mexicana* and *L. brasiliensis* [24, 122–125]. Most of the reports suggest a role for the infection of mammalian host cells, although there is some evidence indicating that receptors at the surface of *L. braziliensis* are able to recognize plasma membrane HS in Lulo cells, which is a cell line derived from *Lutzomyia longipalpis* gut cells [126]. This interaction could cooperate with the lectin-dependent binding of promastigotes promoting adhesion to the sand fly gut.

Receptors of the parasite involved in the recognition of GAGs are collectively called heparin-binding proteins (HPB) although most of them are able to recognize several GAGs, especially highly sulfated heparin-like molecules such as heparan sulfate (HS). They were first enrolled as virulence factors for *L. donovani* parasites, mediating amastigote and promastigote macrophage infections [23–25]. Only stationary-phase promastigotes are able to express these proteins, which is consistent with the presence of infective promastigotes at this phase of the axenic culture [23, 24]. Surprisingly, pre-incubating promastigotes with heparin enhanced macrophage infection suggesting that heparin could provide a bridge between the parasite HPB and macrophage heparin receptors [24].

*Leishmania amazonensis* amastigotes have a high affinity for heparin and especially for HS proteoglycans at the surface of host cells. Mammalian host cells treated with heparitinase lose their ability to bind to *L. amazonensis* amastigotes. Differently to what happens in *L. donovani* infection, addition of exogenous heparin blocked amastigote adherence to non-myeloid mammalian cells such as CHO, HEp-2 and NIH-3T3 cells. In addition, efficient inhibition of amastigote-to-macrophage binding was obtained by pre-treating the parasite prior to their addition to macrophage cultures [125]. However, HS efficiently inhibits *L. amazonensis* amastigote binding to macrophages. Amastigotes are incapable of binding to mutant CHO cells that do not produce GAGs or do not synthesize specifically HS. CHO cells that express undersulfated HS are also less capable of binding to amastigote forms, even when these cells express similar amounts of sulfated proteoglycans due to overexpression of chondroitin sulfate [125]. Amastigotes of *L. major* express a similar HBP, being the receptor specific to HS or heparin-like GAGs [125]. This data is interesting because HS is more relevant for amastigote/macrophage interactions since this GAG is abundant at the membrane of macrophages. However, the effect of HS or heparin blocking macrophage infection is partial (ranging from 40–60%), suggesting cooperation with additional mechanisms for macrophage infection [125]. It was demonstrated that proteins with heparin-affinity were expressed only at the flagellum and the flagellar pocket of *L. donovani* [23] but *L. braziliensis* promastigotes express HBP at the plasma membrane, although HBP is more abundant and have higher activity at the flagellar fraction of the parasite [123, 124]. *Leishmania infantum chagasi* promastigotes express HBP at the plasma membrane, flagellum and flagellar pocket. It seems that these proteins are also stored inside the parasite cell, since they could be detected on several intracellular compartments such as the nucleus, mitochondria and kinetoplast. Polyclonal anti-HBP antibodies were not able to block HBP-dependent infection *in vitro* [123, 124]. This may be due to secondary opsonization. The presence of heparin was able to decrease promastigote internalization by macrophages, but not adhesion [124]. This suggests that for *L. infantum chagasi* HBP may play a role as a phagocytic ligand.

### Glycosaminoglycan-based therapeutic strategies

It is possible that HBP are canonical proteins involved in the invasion of host cells by protozoan parasites, particularly trypanosomatids. *Trypanosoma cruzi*, the etiological agent of Chagas disease expresses a HBP with affinity for HS and heparin. These proteins are expressed by trypomastigote and amastigote forms and are involved with cardiomyocyte infection [127, 128]. It was observed that heparin and HS effectively inhibit cardiomyocyte infection, but not keratan sulfate, dermatan sulfate or *N*-acetylated HS [128], demonstrating the specificity of the interaction, as well as the similarities to the HBP expressed by *Leishmania* parasites. Although several reports demonstrated the relevance of HBP-dependent parasite infection there is no evidence that this host-parasite interplay can be explored as a therapeutic target. HBP-dependent infection *in vitro* is successfully inhibited with HS, heparin and heparin-like molecules, which place these molecules as possible compounds capable to diminish parasite invasion and subsequent infection dissemination. Usually, the activity of GAGs as inhibitors of *Leishmania* infection depends on the number and type of saccharide units, sulfation of the molecule and protein interactions [23, 24, 123, 125]. In addition, it is necessary to evaluate possible adverse effects of HBP natural competitors, such as heparin. The anticoagulant activity of heparin can be detrimental to the host even when administered locally, since it may cause parasite dissemination and extensive bleeding. HS, which has an overall decreased but existent ability to inhibit HBP [125], has other features to be considered, such as cost. One possible alternative is the heparin analogues or heparin-like exogenous molecules derived from non-mammalian sources (Fig. 2). Some of these molecules display less anti-coagulant activity but remain capable to interact with mammalian host cells, including modulating inflammatory response [89, 129]. These molecules could be used as antagonists of HBP, decreasing macrophage invasion and disease progression (Fig. 2). However, the ability of these molecules to bind to parasite HBP still needs to be determined.

## Conclusions

Study of *Leishmania*/glycosaminoglycans/host cell interactions may be important to fully understand parasite infection and dissemination. The characteristics of glycosaminoglycans, especially their low toxicity and availability from non-mammalian sources, plus their promising effects in modulate *in vitro Leishmania* infections compared to the current treatment, make them a potential new therapeutic strategies to control tegumentary leishmaniasis.

## Abbreviations

ADP: adenosine diphosphate
AMP: adenosine monophosphate
ATP: adenosine triphosphate
bFGF: basic fibroblast growth factor
CD: cluster of differentiation
CHO cells: Chinese hamster ovary cells
CPB: cysteine proteinase B
CR: complement receptor
CS: chondroitin sulfate
DS: dermatan sulfate
ECM: extracellular matrix
EGF: epidermal growth factor
GAG: glycosaminoglycan
GalNac: *N*-acetyl-galactosamine
GIPLs: glycoinositolphospholipds
GlcNAc: *N*-acetyl-glucosamine
GNAT: *N*-acetylglucosamine acetyltransferase
gp63: glycoprotein 63
GPI: glycosylphosphatidylinositol
HA: hyaluronic acid
HBP: heparin-binding protein
Hep: heparin
Hep-2 cells: human epithelial type 2 cells
HGF: hepatocyte growth factor
HS: heparan sulfate
IgG: Immunoglobulin G
IL-10: interleucine 10
IL-12: interleucine 12
iNOS: inducible nitric oxide synthase
IX: collagen 2
KS: keratan sulfate
LPG: lipophosphoglycan
MHC: major histocompatibility complex
MR: mannose receptor
NLR: nucleotide-binding oligomerization domain containing 2 (NOD2)-like receptor
NO: nitric oxide
PAMP: pathogen-associated molecular pattern
PG: proteoglycans
PGE_2_: prostaglandin E_2_
PRR: pattern recognition receptor
PS: phosphadidylserine
PSG: promastigote secretory gel
TGF-β: transforming growth factor beta
TLR: toll-like receptor
TNF-α: tumor necrosis factor alpha
VEGF: vascular endothelial growth factor
